# From campus to communities: evaluation of the first UK-based bystander programme for the prevention of domestic violence and abuse in general communities

**DOI:** 10.1186/s12889-020-08519-6

**Published:** 2020-05-13

**Authors:** Alexa N. Gainsbury, Rachel A. Fenton, Cassandra A. Jones

**Affiliations:** 1 University of Exeter, Exeter, EX4 4PY UK; 2 University of Strathclyde, G1 IXQ Glasgow, Scotland

**Keywords:** Bystander, Community, Domestic violence and abuse, Interpersonal violence, Prevention, Violence against women and girls

## Abstract

**Background:**

Violence against women and girls is a public health epidemic. Campus-based research has found bystander programmes show promise as effective primary prevention of sexual violence. However, evidence regarding domestic violence and abuse bystander prevention specifically, and in community settings generally, is still in development. Further, research has predominantly emanated from the US. Examining proof of concept in differing cultural contexts is required. This study evaluates the feasibility and potential for effectiveness of a domestic violence and abuse bystander intervention within UK general communities—Active Bystander Communities.

**Methods:**

Participants recruited opportunistically attended a three-session programme facilitated by experts in the field. Programme feasibility was measured using participant attendance and feedback across nine learning objectives. Myth acceptance, bystander efficacy, behavioural intent and bystander behaviours were assessed using validated scales at baseline, post-intervention, and four-month follow-up. Results were examined for potential backlash. Analyses used a paired sample *t*-test and effect size was quantified with Cohen’s *d*.

**Results:**

58/70 participants attended all programme sessions. Participant feedback consistently rated the programme highly and significant change (*p* ≤ 0·001) was observed in the desired direction across behavioural intent, bystander efficacy, and myth acceptance scores at post and follow-up. Effect size was generally large and, with the exception of *Perception of Peer Myth Acceptance,* improved at follow-up. Backlash was minimal.

**Conclusions:**

To our knowledge this is the first UK-based study to examine the potential of bystander intervention as a community-level intervention for domestic violence and abuse. Findings are promising and indicate the translatability of the bystander approach to domestic violence and abuse prevention as well as community contexts. This is likely to be of great interest to policymakers and may help shape future community-based interventions. Further research is now needed using experimental designs engaging diverse community audiences.

## Background

Violence against women and girls (VAWG) is a public health crisis of ‘epidemic proportions’ [1] (p3) which impacts severely upon individuals and communities. The burden that VAWG places on health, social care and justice resources [2] is so great “that even marginally effective interventions are cost effective” [3]. Whilst legislative responses to VAWG have been historically piecemeal, one positive UK advancement is the Consultation Response and Domestic Abuse Draft Bill 2019 [4]. In addition to enhancing justice and survivor processes, the Consultation highlights the need for raising awareness and changing social attitudes which are supportive of violence. However, juxtaposed with this policy development, is a popular culture in which UK media narratives recently attempted to silence and de-legitimise neighbours for alerting police in the case of suspected abuse in the home of the now British Prime Minister [5, 6].

One way of moving social attitudes might be through bystander programmes, which show promise as effective primary prevention of VAWG [7–10]. Bystander approaches are complex models which seek to engage those outside the victim - perpetrator relationship to play an active role in preventing and responding to VAWG by shifting gender inequitable attitudes, beliefs and cultural norms which support abuse, and ultimately increasing pro-social bystander behaviour to prevent it. As interventions are made, over time social attitudes regarding the acceptability of both VAWG within society and bystander actions will shift [11].

The organising framework for bystander programmes is underpinned by Latané’s Five Step Model of Helping [12]. The model is based on progressing participants through the processes of change; from noticing the behaviour, seeing it as a problem, assuming responsibility for helping and possessing the skills for effective and safe intervention, through to the final stage of taking action. Further, bystander programmes also aim to deliver changes in attitudes and beliefs, and social and cultural norms which are associated with enhanced bystander likelihood and reduced perpetration, such as sexism, empathy and rape myth acceptance [7]. The incorporation of social norms theory is thought to be maximally effective because peer norms are variables for bystander intervention [7]: perceptions of others’ willingness to intervene is related to bystander behaviour [13]. Peer norms are also related to perpetration where they are supportive of abuse [14–16]. Consequently, correcting misperceptions about others’ intentions to intervene and support for abusive behaviours may ameliorate barriers to intervention [7, 14, 15], although studies have rarely reported on peer norm perceptions [7].

As a reduction in incidence of violence is problematic to measure, most studies use proxy outcome measures which correlate with the aims and determinants of bystander programme effectiveness [7]. Thus, studies, including experimental, have found significant change in the desired direction in victimisation and perpetration at a community level, bystander attitudes, efficacy and intent, rape myth rejection, knowledge and empathy, and actual bystander behaviours. The evidence is available elsewhere [7–10, 17]. Law plays a symbolic role in communicating the acceptability of behaviours to the public and is central to combatting VAWG at a structural level [18]. Many bystander programmes intend to increase knowledge on substantive law but this is rarely reported on [7].

There are a number of widely accepted criteria for effective prevention programming for behavioural change [7, 19, 20]. Bystander programmes should be underpinned by theory and evaluated accordingly. Measurement should include potential backlash effects, as some prevention efforts may have the opposite outcome to that intended [7], such as an entrenchment of the attitudes programming is attempting to shift [21]. Further, as permanency of outcomes is uncertain [10] and programme effects may diminish over time [8], follow-up is important. Other criteria relate to effective pedagogy, design and implementation [20, 22]. Longer programmes which are cumulative, sequential and delivered over time by well-trained facilitators are more effective [10, 20, 23]. A wide range of teaching pedagogies including emphasis on role-play for skills acquisition and use of socio-culturally relevant materials are indicated [10, 20]. Mixed- sex groups are also appropriate for bystander programmes [10, 24].

The research base for bystander programmes as primary prevention of domestic and sexual violence and abuse (DSVA) emanates predominantly from the US with a focus on campus sexual violence prevention. Although there is some evidence as to the translatability to other non-college audiences in the US [10]. To date, little is known about domestic violence and abuse (DVA) bystander programmes and general communities. However, we do know that VAWG is pervasive [1] and that third parties may both witness warning signs, incidents of abuse [25, 26] and be in a position to help, and that abusers misperceive norms about others’ use of DVA [27]. Thus, understanding the applicability, utility and limitations of bystander programmes in UK non-student contexts and as a DVA prevention tool is an important next step. Compared to university populations, general communities represent a challenge in terms of implementation. Universities have a strategic interest in prevention, a captive audience with a potential shared identity as a student of that institution, and in situ environmental space for delivery and the making of supported interventions. These factors are absent for general communities, resulting in complexities in determining commonalities of potential audience both in terms of physically bringing people together to receive an intervention and in terms of fostering communities for making supported interventions [28, 29]. Capturing the acceptability of programme content, implementation and delivery approaches within the community context is thus important.

This paper reports on the quantitative phase of a mixed methods feasibility study [30] intended to determine the acceptability and potential utility of the first UK DVA bystander intervention within general communities: Active Bystander Communities (ABC). The study involved a pre-post and follow-up survey design, reported on in this paper. We also undertook 17 semi-structured interviews with participants, approximately 1 month after the intervention, which examined the processes that lead to change and how they map onto the theoretical underpinnings of the programme. These are reported on elsewhere [31]. In this paper we contribute to the developing evidence base regarding the translatability of bystander interventions to DVA prevention and make a significant contribution to exploring the proof of concept for the first time within broader societal contexts.

## Methods

### Study population

The study was conducted across three local authority areas in the South West of England. Participants over 16 who identified as a community member or undertook a community-facing role (professional or volunteer) were recruited opportunistically using snowballing over the 2 month period prior to intervention delivery in February 2019; information promoting the pilot and the opportunity to attend ABC was disseminated to community-facing organisations and groups via community and professional networks (emails and face-to-face). A booking link was also featured on an online hub for local community organisations and paper fliers distributed to community organisations (for example churches and charity hubs) within one area of Exeter. An email accompanying project information encouraged participants to pass information onto interested parties and indicated we were particularly interested in engaging men. We also reached out directly to two large predominantly male community groups in the local area to encourage their participation. Because of the nature of snowballing we were unable to track either the reach or response rate our recruitment method elicited. Participants were arranged into five pilot groups according to geography and session time preference (morning, afternoon or evening), with a maximum of 20 participants per group, thought to be optimal based on author and facilitator experience (Additional file 1).

### The intervention

ABC was co-created by researchers and DVA and public health practitioners [28]. The theoretical design, content and pedagogy was adapted from *The Intervention Initiative* [32]. Structurally and theoretically ABC follows *The Intervention Initiative* design [7, 19, 22, 28, 29] and progresses participants through Latané’s five step theoretical model [12] over three sessions. Sessions one and two correspond with the first three stages for intervention (noticing to responsibility) and session three corresponds with the skills training in stage four [19, 28, 29]. In accordance with the criteria for effective prevention [20], varied pedagogy was utilised, including presentation, media, active learning exercises, group work and role-play vignettes. Content and the process of co-creation is described in detail by Fenton et al. 2019 [28]. In brief, content was adapted to use information on DVA prevalence, impacts and myths, and the law relating to DVA. All examples in the presentation and group exercises were changed to be proximal and salient to DVA in adult general populations (for example references to campus resources were changed to community resources; scenarios were changed to encompass family situations including children, rather than student bar-based or classroom contexts). New DVA role-plays were designed and scripted by a DVA specialist agency and included appropriate responses to perpetrators and victims [28] as well as community sources of support related to DVA agencies. Participants received handouts containing a summary of the information delivered within the intervention and slides. In accordance with best practice [20], ABC was delivered to four groups of between nine and 20 participants over 3 two-hour sessions 1 week apart by two expert co-facilitators, one male and one female, one of whom was a specialist from a DVA service provider. In order to compare feasibility, one further group of 16 was arranged to receive the same intervention over the course of a day. Participant wellbeing was addressed at the start of each session and community sources of DVA specialist support reiterated. If a participant needed support during or after a session this was provided by the DVA specialist facilitator.

### Procedure

Facilitators collected attendance data. Participants completed paper-based questionnaires before session one began and after session three finished, and an online survey at four-months post-intervention. The questionnaire administered at programme end contained course evaluation feedback questions. Written informed consent was given and participants could still attend the intervention if they did not consent to participate in the research. Only participants who attended the full programme were included in the post and 4 month follow-up analysis.

One pilot group of participants (*n* = 9) who received the intervention approximately 1 year in advance of the substantive study were not asked to complete the four-month follow-up for practical reasons.

Participants received a £5 e-voucher after completing the post-intervention survey. Ethical approval was given by the College of Social Sciences and International Studies Ethics Committee at the University of Exeter.

### Measures

#### Attendance

Facilitators registered attendance at each session.

#### Participant demographics

Participants were asked to describe their age, gender, sexual orientation, ethnicity and whether English was their first language at baseline. Participants were also asked to write down (open text) their motivations for attending, whether they knew someone who had been affected by domestic abuse (yes, no, not sure), if they had attended a programme about domestic abuse (in the last 5 years or ever) and if they had taken part in a campaign that raises awareness about domestic abuse (in the last 5 years or ever).

#### Programme feedback

Participants were asked to rank the programme on a five-point rating scale with one being “definitely no” to five being “definitely yes”, against nine learning objectives (Table 1).

### Instruments

Part of the purpose of this feasibility study was to assess the utility of instruments. The instruments adopted correspond to the theoretical processes of change for bystander action commonly reported on in the extant literature relating to bystander intent, efficacy and behaviour. Whilst these measures originate in US college-based sexual violence research we theorised that they would be equally applicable because the processes for change are likely to remain the same, and because they are likely translatable to other forms of violence [10]. Thus, we adapted the content of these measures from sexual violence to DVA, and in accordance with previous work, altered the language to UK-English and UK concepts [19, 22, 29]. We provide examples of these adaptions below. Most bystander evaluations have used rape myth acceptance [7], and, accordingly, we changed our attitudinal measure to domestic abuse myth acceptance. Regarding peer norms, we changed the comparator group from “people in your peer group (other students of the same sex as you at this university)”, to “friends, family and neighbours of the same gender” to correspond with community participants. A participant mean score was calculated for each instrument based on their responses to items within that scale.

#### DVA myth acceptance

We used the Domestic Violence Myth Acceptance Scale [33] (DVMAS) to measures attitudes and beliefs about DVA which includes items about prevalence (“Domestic abuse does not affect many people”) as well as attitudes towards victimhood and perpetration (“If a woman continues living with a man who beats her then it’s her own fault if she gets beaten again”). We asked participants to indicate their level of agreement with 16 described statements on a scale of one (strong disagreement, i.e. myth rejection) to seven (strong agreement, i.e. myth endorsement).

#### Bystander efficacy

We used a shortened adapted version of Banyard et al’s (2005) Confidence Scale [34]. Examples of amendments and items include “Call for help (I.e. call 999) if I hear someone in my neighbourhood yelling ‘help’” and “Expressing my discomfort if someone says that domestic abuse victims are to blame for being abused” (adapted from “Call for help (I.e. call 911) if I hear someone in my dorm yelling ‘help’” and “Expressing my discomfort if someone says that rape victims are to blame for being raped”. We asked participants to score their degree of confidence in enacting 11 described behaviours from 0% (no confidence) to 100% (full confidence).

#### Bystander intent

We used items from the Bystander Attitude-Scale Revised and Bystander Behavior Scale Revised [35] alongside Intent to Help Scales [36] to measure participants’ attitudes and likelihood of helping others. Example items include “Speak up to someone who is calling his/her partner names or swearing at them” and “Approach someone I know if I thought they were in an abusive relationship and let them know I’m here to help”. Participants indicated their likelihood of taking 17 described actions on a scale of one (not at all likely) to five (extremely likely).

#### Bystander behaviours

We used the Bystander Behavior Scale – Revised (BBS-R) (as modified by McMahon et al. 2011 [37]) to measure behaviours participants had recently engaged in. Participants were asked to think about the previous 2 months for the pre-intervention survey and “since attending” the intervention for the post-intervention and follow-up surveys. They reported “Yes”, they engaged in that behaviour (1); “No”, they did not engage when in the situation (− 1); or they were “Not in the situation” (0) for 19 described scenarios. Items included “Went with a friend, relative, or neighbour to the police to be of support as they filed a complaint related to domestic abuse, e.g. restraining order” and “Visited a website to learn more about domestic abuse” (adapted from “Go with a female friend to the police department if she says she was raped” and “Visit a website to learn more about sexual violence”. Composite scores were calculated by summing the score on each item. We also report on the mean number of times participants recognised themselves as being in the situation described as identified by either a score or 1 (“Yes”) or − 1 (“No”).

#### Perception of peer DVA myth acceptance

We used a subset of DVMAS items included in our *DVA Myth Acceptance* scale. Participants indicated what proportion (0–100%) of their friends, family and neighbours of the same gender they thought would agree with four described statements.

#### Perception of peer behavioural intent

We used a subset of items included in our *Behavioural Intent* scale and asked participants to indicate how likely they thought that friends, family and neighbours of the same gender would enact six stated behaviours on a scale of one (not at all likely) to five (extremely likely).

#### Perception of law knowledge

We asked participants to rate their overall knowledge about law relating to DVA on a scale of one (very poor) to five (very good).

#### Backlash

We followed Moynihan (2011) [38] and calculated the difference between participants’ pre and post mean scores for the scales *Behavioural Intent* and *Myth Acceptance*. An attitudinal change in the undesired direction ≥1 standard deviation (SD) from the study population was taken to indicate a substantial negative shift that could be attributable to backlash.

### Statistical analysis

StataSE 15 [39] was used to undertake data analysis. We generated descriptive statistics for attendance, participant demographics and programme feedback. We calculated paired sample *t*-tests and 95% confidence intervals for scale aggregate means at pre, post-intervention and follow-up for the scales examining *DVA Myth Acceptance* (self and perception of peers), *Bystander Efficacy, Behavioural Intent* (self and perception of peers), *Bystander Behaviours* and *Perceived Law Knowledge*. We examined paired data at each stage so as not to present artificially high difference between pre and post-intervention measures. Effect size was quantified using Cohen’s *d* (*d* > 0.8 is indicative of a large effect whilst *d* > 0.5 is viewed as the minimum threshold for meaningful change [40].

## Results

**Fig. 1:**
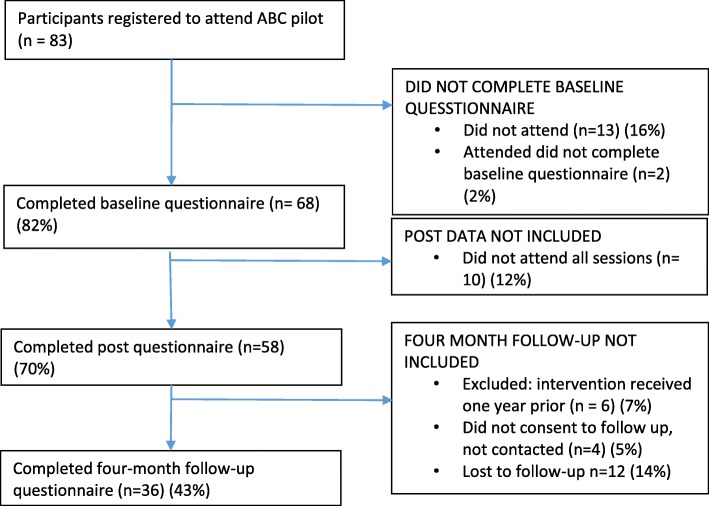
Participant flow

Our recruitment method resulted in 83 people registering to attend ABC of which 70 (84%) attended and 68 (82%) participated in the study. 67/70 (96%) attended two sessions and 58/70 (83%) attended all three sessions. Figure 1 describes participant flow through the study. Attrition from registration to attendance was greatest amongst those booked onto the full day session: 10/16 (63%) of registrants did not attend the full day session compared to 3/67 (4%) booked onto a three-session programme. Attrition within programme was greatest amongst participants attending an evening programme; 17/25 (68%) of evening (6-8 pm or 7-9 pm) participants attended all three sessions compared to 35/39 (90%) of those attending a day programme (10 am-12 pm or 2 pm–4 pm). Overall 58/70 (89%) participants received the full intervention dose and completed pre and post questionnaires, and 36/52 (69%) of eligible participants received the full intervention dose and completed four-month follow-up.

### Participant demographics (Additional file 2)

Eighteen (26%) of participants identified as male and 1 (1%) as a transgender man (included in analysis as male). Nearly all (93%) identified as heterosexual and white British (96%) with English as their first language (97%). Participant age ranged from 16 to 73 years. 49/68 (72%) identified as being in a relationship. 48/60 (47% of men and 80% of women) knew someone who had been affected by domestic abuse. Most had not previously attended domestic abuse training nor been involved in a campaign raising awareness of domestic abuse although women were more likely to have done so than men. As motivations for attending were collected qualitatively, no descriptive data was generated. However, the most common theme was supporting participants’ occupations (paid or voluntary) with approximately half identifying this as a reason for attending.

### Programme feedback

The programme was very well-received and participants’ (*n* = 58) self-reported learning measures consistently scored > 4 out of a possible five (Table 1). There was some difference in feedback between genders, however, due to small sample size this was rarely significant.

**Table 1:** Mean participant feedback score against learning objective (out of a possible 5)

The programme met its objectives in assisting me to:	Mean Score (95% CI)	Male (95% CI)	Female (95% CI)
Improve my knowledge about domestic abuse	4.6 (4.4–4.8)	4.9 (4.7–5.0)	4.4 (4.2–4.7)
Understand that domestic abuse is a serious problem in society	4.7 (4.6–4.9	4.7 (4.5–5.0)	4.7 (4.5–5.0)
Understand that coercive control is a criminal offence	4.5 (4.3–4.8)	4.6 (4.3–4.9)	4.5 (4.2–4.8)
Know where to go for help and or support in cases of domestic abuse	4.3 (4.0–4.5)	4.1 (3.8–4.5)	4.3 (4.0–4.6)
Understand the stages of bystander interventions from noticing to acting	4.7 (4.5–4.8)	4.4 (4.0–4.8)	4.8 (4.6–4.9)
Understand that individuals can often be mistaken about others’ beliefs and values	4.4 (4.2–4.6)	4.5 (4.1–4.9	4.4 (4.1–4.7)
Be familiar with intervention strategie	4.5 (4.4–4.7)	4.3 (4.1–4.6)	4.6 (4.4–4.8)
Be confident to use intervention strategies in your everyday life	4.2 (4.1–4.4)	4.0 (3.7–4.3)	4.3 (4.1–4.5)
Increase the likelihood you will use intervention strategies in your everyday life	4.4 (4.2–4.6)	4.0 (3.7–4.3)	4.6 (4.4–4.8)

### Utility of instruments

Scale reliability (Cronbach’s α) was good or acceptable at each stage (Table 2), except for *Perception of Peer Myth Acceptance* at four-month follow-up. A small number of participants fedback (either verbally during completion or by annotating their surveys) difficulty in assigning a single score to family, friends and neighbours due to perceived differences between these groups.

**Table 2:** Paired two-tailed *t*-test, effect size (Cohen’s *d)* and scale reliability (Cronbach’s α) post-intervention and follow-up

Measure	Unpaired Pre-test (***n*** = 68)	Paired Pre-test (***n*** = 58)	Post-test (***n*** = 58)				Four-month follow-up (***n*** = 36)^**a**^			
	Mean (CI) [α]	Mean (CI)	Mean (CI)	Change from pre (CI)	P	*d*	Mean (CI)	Change from pre (CI)	P	*d*
*DVA Myth Acceptance (1 = strongly disagree, 7 = strongly agree)*	**2.09** (1.95–2.23) [0.56]	**1.97** (1.81–2.13) [0.67]	**1.50** (1.38–1.61) [0.71]	**−0.48** (− 0.28–0.67)	< 0.0001	0.83	**1.45** (1.28–1.63) [0.78]	**0.52** (0.29–0.75)	0.0001	1.02
*Bystander Efficacy (0–100%)*	**79.88** (76.51–83.26) [0.83]	**81.07** (77.59–84.55) [0.87]	**91.37** (89.14–93.59) [0.85]	**10.30** (6.21–14.38) [0.86]	< 0.0001	0.93	**93.65** (91.29–96.00) [0.77]	**12.01** (7.17–17.02)	< 0.0001	1.15
*Behavioural Intent (1 = not at all likely, 5 = very likely)*	**3.97** (3.84–4.11) [0.71]	**4.03** (3.89–4.17) [0.86]	**4.42** (4.32–4.52) [0.81]	**0.40** (0.23–0.57)	< 0.0001	0.86	**4.44** (4.35–4.52) [0.58]	**0.44** (0.26–0.62)	< 0.0001	1.16
*Perception of Peers’ DVA Myth Acceptance (% who would agree with statement)*	**35.39**^**b**^ (31.20–39.57) [0.61]	**34.54**^**c**^ (30.11–38.97) [0.66]	**23.65** (19.13–28.16) [0.75]	**- 10.89** (4.63–17.16)	0.0008	0.65	**28.30** (26.52–35.47) [0.30]	**−5.55** (−14.47–3.37)	0.22	0.30
*Perception of Peers’ Behavioural Intent (1 = not at all likely, 5 = very likely)*	**3.68** (3.52–3.84) [0.78]	**3.66** (3.49–3.83) [0.80]	**4.02** (3.86–4.18) [0.87]	**0.36** (0.13–0.60)	0.0023	0.58	**4.19** (3.96–4.42) [0.89]	**0.54** (0.31–0.75)	0.0012	0.79
*Bystander Behaviours (1 took action, 0 not in situation, −1 didn’t take action)*	**0.74** (− 0.39–1.87) [0.74]	**0.88**^**d**^ (− 0.35–22) [0.69]	**1.41**^**d**^ (0.23–2.80) [0.79]	**0.53** (−1.31–2.37)	0.57	0.11	**2.16**^**e**^ (0.57–3.76) [0.71]	**1.17** (− 0.84–3.17)	0.25	0.27
*Number of listed bystander scenarios experienced (*i.e. *scored either 1 or − 1)*	**8.24** (7.04–9.43)	**7.92**^**d**^ (6.56–9.28)	**7.94**^**d**^ (6.76–8.82)	**0.02** (− 1.76–1.80)	0.98		**8.89**^**e**^ (6.14–8.94)	**1.35** (− 0.62–3.31)	0.18	
*Perceived Law Knowledge (1 = very poor, 5 = very good)*	**2.75** (2.50–3.00)	**2.79** (2.52–3.06)	**3.67** (3.48–3.87)	**0.89** (0.66–1.11)	< 0.0001	0.99	**3.92** (3.70–4.14)	**1.01** (0.65–1.45)	< 0.0001	1.24

^a^Paired data only

^b^*n* = 65 due to blank responses at baseline

^c^*n* = 55 due to blank responses at baseline

^d^*n* = 51 due to six participants excluded from post analysis (received intervention within a day) and one blank response at post

^e^*n* = 35 due to one blank response at follow up

### Effectiveness

We observed a statistically significant change (*p* < 0.01) in the desired direction across *Myth Acceptance* (self and perception of peers), *Bystander Efficacy*, *Behavioural Intent* (self and perception of peers) and *Perceived Law Knowledge* at post. Significance was maintained at four-months with the exception of *Perception of Peer Myth Acceptance* (*p* = 0.22) (Table 2). We observed a change in the desired direction for *Bystander Behaviours* at 4 months follow-up and at post, however this was not statistically significant. Where significant change was observed, effect size was generally large and, with the exception of *Perception of Peer Myth Acceptance*, improved at four-month follow-up.

Male participants had higher *Myth Acceptance* and lower *Bystander Efficacy, Behavioural Intent* and *Bystander Behaviours* scores at baseline and experienced greater change across these measures at post and four-months after the intervention. However, whilst the difference in observed change between genders at baseline was significant, due to wide confidence intervals, the difference in change from baseline was not.

### Backlash

**Fig. 2:**
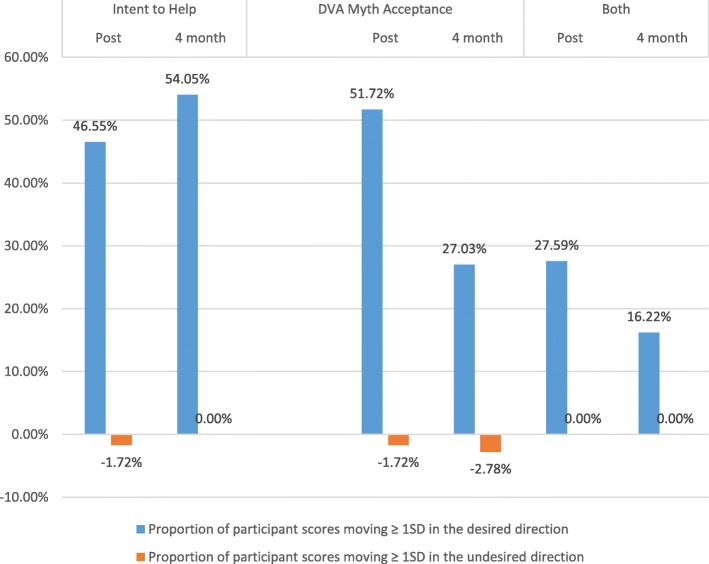
Changes in attitudinal scores ≥1 SD from pre-test to post-test and pre-test to four-month follow-up

We observed backlash in 3% of the study population for *Myth Acceptance* and 2% of the study population for *Behavioural Intent.* This, however, is vastly outweighed by the proportion of participants whose scores improved by ≥1 standard deviation for each of the attitudinal measures at post and follow-up (Fig. 2).

## Discussion

This study was as an explorative descriptive study using a cohort of participants who were the first to undertake a newly developed bystander programme for UK general communities. To our knowledge, it is the first UK-based study to examine the potential of bystander intervention as a community-level intervention for DVA. Findings are very promising and consistent with extant studies, suggesting translatability of the bystander approach to broader societal contexts, including outside North America, and the feasibility and potential for effectiveness of the ABC programme as community-level primary prevention. Further positives are that high effect size was observed even with a self-selecting group with desirable baseline scores, and that improvement was not only maintained but improved upon at follow-up across most measures.

The sustained high attendance over time and positive programme feedback endorses both the pedagogical content and mode of delivery of ABC. Interestingly, 10/16 registered participants for the one-day programme did not attend, compared to 3/67 for the three-session delivery (Additional file 1/Appendix A). Further exploration is required but this observation is counter to assumptions that delivering interventions over multiple sessions risks programme feasibility. Attrition across the programme was low overall, but highest amongst evening attendees when it is reasonable to assume participants were more likely to be attending in their own time. These findings of feasibility warrant further investigation with a larger sample as they are central to future implementation of community-level bystander interventions and sustainability of programmes such as ABC.

Significant change in the desired direction was achieved for participant *DVA Myth Acceptance* (self), *Bystander Efficacy* and *Behavioural Intent* (self and perception of peers) and *Perceived Law Knowledge* at post and follow-up. These findings map positively onto the theoretical model design underpinning ABC which intends to progress participants through the stages of the Model of Helping [12], including changes to participant attitudes and beliefs, perceptions of peer norms and perceived knowledge of law. Given that changes in *Perception of Peers’ DVA Myth Acceptance* were still improved at follow-up, the loss of significance is not suggestive of anything more complex at play and is more likely a result of low power. Whilst numerous campus-based studies [15] have found positive effects decline over time, this study found changes were not only maintained but, with the exception of *Perception of Peers’ DVA Myth Acceptance,* improved upon at four-months, indicating a sustained shift in the variables associated with positive helping behaviours. Further research should explore whether our findings are an effect of participant bias or can be replicated in experimental studies and, if so, examine the features of a community-based intervention that better lends itself to sustained impact than has been previously observed in campus-based studies.

Change in the desired direction was observed for *Bystander Behaviours* at post, and even more so at follow-up. Although these changes were not statistically significant, it is nonetheless promising to witness more bystander behaviours at follow-up when participants had had more time and opportunity to enact interventions [29]. However, it is noteworthy that, even at follow-up, participants had experienced fewer than half the situations described in the scale, limiting opportunity to detect programme effect. Thus, whilst we concur with Jouriles et al. (2018); that small effects observed at individual level can accumulate resulting in real-life impact at societal level [8], we also note that our method of measuring *Bystander Behaviours* may have led to an underrepresentation of enacted behaviours. Rather than asking participants to indicate action against a limited list of pre-defined situations, we suggest the inductive approach of qualitative methods may be best suited to capturing and understanding the breadth and scope of actual bystander behaviours. These insights may subsequently be helpful for the development of tools specific to DVA community intervention to capture behaviours in future studies. Our findings on backlash compare well with other studies [29] and backlash appears minimal.

With the exception of *Bystander Behaviours,* measures adapted for DVA appeared appropriate to the study design, mapping onto previous findings [22]. However, we note the difficulty of establishing a peer group comparator for individuals who come together randomly as opposed to in a defined peer setting such as a university cohort. Although the findings suggest acceptable scale reliability, with the exception of *Peers’ DVA Myth Acceptance,* the use of the “friends, family and neighbours” needs to be examined further.

### Limitations

These findings should be read within the context of several limitations. Resource and practicalities precluded both an experimental design and identifying a matched control group of sufficient size to enable meaningful comparison. Whilst attrition was low and completeness of questionnaires generally good, we cannot rule out the potential bias arising from missing data and study design, including a convenience sample, cannot preclude the potential for participant bias. Thus, promising findings should not overshadow the potential for false significant results. Sub-group analysis, including difference in the potential effectiveness of a day programme compared to a three-session programme, was prevented by the small sample size and the predominance of white heterosexual women participants. Despite our recruitment strategy focusing on encouraging male attendance, we achieved only 26% men. Considering the gendered nature of VAWG, and indications that bystander interventions may be particularly effective in engaging men [7, 41–43], this is disappointing but speaks to the broader challenge of engaging men in DVA prevention [44, 45].

Our recruitment method resulted in a self-selecting cohort; whilst most had not previously attended domestic abuse training, nor been involved in domestic abuse awareness campaigns, baseline scores were desirable and around half identified occupational (voluntary and paid) reasons for attending, suggesting a highly engaged sample who may have been more receptive to the intervention. It is still promising to observe positive findings amongst a ‘warm’ cohort as they may be well placed to enact interventions within personal and professional spheres. However, it is important to note that the intervention remains untested amongst broader populations. Recruiting diverse samples (in terms of demographics and pre-existing level of engagement) is a challenge, particularly at pilot stage where resource is small and programme feasibility and potential for effectiveness is still unknown. In this context communities are different to the college campuses previous studies have recruited from. Universities, for example, can encourage attendance by positioning bystander programmes as mandatory learning modules whereas community focused programmes need alternative ways of engaging diverse audiences for both evaluation and implementation. This itself needs further study but should include consulting with communities to identify routes in, as well as developing approaches identified by existing literature such as utilising existing social networks [45]. Exploring the workplace as an intervention setting or the feasibility of integrating interventions within existing community infrastructure may also be beneficial.

Finally, the decision to collect four-month data electronically was a practical one and, whilst consistency was observed, electronic administration may have affected responses. The inclusion of follow-up data is a strength of the study as there is a paucity of evidence regarding the positive lasting impact of interventions, vital in the context of real-world application. However, the timeframe is still limited, not sufficient to advance our understanding of the potential longer lasting impacts of bystander interventions and may have limited the opportunity to collect bystander behaviours enacted over time.

## Conclusions

This feasibility study makes a significant and timely contribution to the emergent evidence base on bystander interventions in the UK context. It indicates the transferability of the bystander approach to violence prevention from student populations to general communities, and from sexual violence to DVA. Our study is the first UK community-based study to show feasibility and significant effects on variables associated with positive helping behaviours and supports the hypothesis that bystander interventions can be a potentially effective strategic component of community-level primary prevention of DVA. Bystander programmes such as ABC may therefore be an important vehicle for the awareness-raising and changing of social attitudes and norms, foreseen as necessary by the Home Office Domestic Abuse Consultation Response [4], but not actually provided for in practice [4].

More research is now required using experimental designs and diverse community audiences. To further understand feasibility, acceptability and effectiveness, as well as any implications as to health inequalities, it is vital to understand if and how we can engage diverse populations and the impact that audience has on outcomes. Future studies should focus on the under-studied issue of recruiting harder-to-reach populations and those with no prior understanding or engagement with DVA prevention.

More research is also needed as to how best to describe peers within the community context as well as capture bystander behaviours and, considering the limitations of quantitative methods in relation to unknown outcomes, the potential use of qualitative methodologies. Further examination of the interaction between perceived knowledge of law and the processes and variables leading to change is required to understand the role of law, if any, within bystander interventions.

## Supplementary information

**Additional file 1.** “Group Composition by number of sessions attended (and gender)”. The table provides details of participant attendance by group gender and session.

**Additional file 2.** “Participant demographics and prior experience of domestic abuse provided at baseline”. The table provides details of participants’ demographics and self-reported experience of attending previous domestic abuse training or participating in domestic abuse campaigns.

## Data Availability

The datasets used during the current study are available from the corresponding author under reasonable request.

## Abbreviations

ABC: Active Bystander Communities
DVA: Domestic Violence and Abuse
DVMAS: Domestic Violence Myth Acceptance Scale
US: United States
VAWG: Violence Against Women and Girls
