# Supplementary material for: From campus to communities: evaluation of the first UK-based bystander programme for the prevention of domestic violence and abuse in general communities
Source: BMC Public Health. 2020 May 13;20:674. doi: 10.1186/s12889-020-08519-6 (PMC7218832; doi:10.1186/s12889-020-08519-6)
Supplement: Supplementary file 1 — Additional file 1. “Group Composition by number of sessions attended (and gender)”. The table provides details of participant attendance by group gender and session. [file 12889_2020_8519_MOESM1_ESM.docx]

**Group composition by number of sessions attended (and gender).**

| Group | Booked to attend (capacity) (gender) | Attended 1 Session  (gender) | Attended 2 Sessions  (gender) | Attended 3 sessions  (gender) | Format |
| --- | --- | --- | --- | --- | --- |
| Cheltenham  7-9pm | 9  (8f, 1m) | 9  (8f, 1m) | 9  (8f, 1m) | 6  (6f, 1m) | 3 * 2 hour sessions held one week apart. |
| Exeter 1  2pm-4pm | 20 (20)  (13f, 7m) | 20  (13f, 7 m) | 20  (12f, 7m) | 19   (12 f, 7m) | 3 * 2 hour sessions held one week apart. |
| Exeter 2  6pm-8pm | 19 (20)  (16f, 3m) | 16  (14f, 2m) | 13  (13f, 1m) | 11  (10f, 1m) | 3 * 2 hour sessions held one week apart. |
| Torquay  10am-12pm | 19 (20)  (10f, 9m) | 19  (10f, 9m) | 19  (10f, 9m) | 16  (9f, 7m) | 3 * 2 hour sessions held one week apart. |
| Exeter 3  9am-4:30 pm | 16 (16)*  (16 f) | 6 (6f) | | | 3 * 2 hour sessions delivered over one day. |
| Total | 83  (63f, 20m) | 70  (51f, 19m) | 67^e^^[[1]](#footnote-1)^  (49f, 18m) | 58^e^5  (43f, 17m) |  |

*group capacity limited due to room size.

1. e Number includes the six who attended three sessions across one day  [↑](#footnote-ref-1)
