# Supplementary material for: From campus to communities: evaluation of the first UK-based bystander programme for the prevention of domestic violence and abuse in general communities
Source: BMC Public Health. 2020 May 13;20:674. doi: 10.1186/s12889-020-08519-6 (PMC7218832; doi:10.1186/s12889-020-08519-6)
Supplement: Supplementary file 2 — Additional file 2. “Participant demographics and prior experience of domestic abuse provided at baseline”. The table provides details of participants’ demographics and self-reported experience of attending previous domestic abuse training or participating in domestic abuse campaigns. [file 12889_2020_8519_MOESM2_ESM.docx]

**Additional File 2: Participant demographics and prior experience of domestic abuse provided at baseline**

|  | Male (n=19)* | Female (n=49) |
| --- | --- | --- |
| Demographics | | |
| Age | 17-72 (mean 41.4) | 16-73 (mean 40.3) |
| White British | 20 (100%) | 46 (94%) |
| Heterosexual | 18 (95%) | 44 (92%) |
| English first language | 20 (100%) | 47 (96%) |
| Currently in a relationship | 15 (79%) | 34 (69%) |
| Relevant Experience | | |
| *Do you know someone who has been affected by domestic abuse?*  Yes  No  Unsure | 9 (47%)  6 (32%)  4 (21%) | 39 (80%)  4 (8%)  6 (12%) |
| *Have you done a programme to learn about domestic abuse in the last 5 years, i.e. between 2014 and 2019?*  Yes  No  Unsure | 3 (16%)  15 (79%)  1 (5%) | 16 (33%)  29 (59%)  4 (8%) |
| *Have you done a programme before 2014 to learn about domestic abuse*  Yes  No  Unsure | 0  18 (95%)  2 (5%) | 8 (16%)  38 (79%)  3 (6%) |
| *Have you taken part in a campaign that raises awareness about domestic abuse in the last 5 years, i.e. between 2014 and 2019?*  Yes  No  Unsure | 3 (16%)  12 (63%)  4 (22%) | 15 (31%)  33 (67%)  1 (2%) |
| *Have you taken part in a campaign that raises awareness about domestic abuse before 2014?*  Yes  No  Unsure | 18 (95%)  1 (5%) | 3 (6%)  44 (90%)  2 (4%) |

*includes 1 transgender male
